# Stenting of critical aortic coarctation in neonates between 600 and 1,350 g. Using a transfemoral artery approach. A single center experience

**DOI:** 10.3389/fcvm.2022.1025411

**Published:** 2022-10-03

**Authors:** Nathalie Mini, Peter A. Zartner, Martin B. E. Schneider

**Affiliations:** Department of Cardiology, Paediatric Heart Centre, University Hospital of Bonn, Bonn, Germany

**Keywords:** aortic coarctation, very low weight birth infants, cardiac catheter interventional treatment, echocardiography-guidance, extremely low weight infants

## Abstract

**Background:**

Stenting of aortic coarctation (CoA) in newborns with a very low bodyweight remains rare and challenging. In this study we aim to highlight on two points: first the feasibility of CoA stenting in such babies and second the importance of using echocardiogram for guiding the intervention without the need for contrast agent.

**Methods:**

Between 2020 and 2022 three preterm babies with very low (VLWB) and extremely low weight (ELWB) underwent CoA-stenting in our center. The weight of the patients at time of intervention was 1,350, 1,200, and 600 g, respectively. The femoral artery was chosen in all patients as vascular access. Transthoracic echocardiography, sonography of the femoral arteries and head ultrasound were applied for follow up.

**Results:**

All three interventions were successfully done, with no complications. Coronary stents were implanted. In one Patient (1,350 g) the stent was inserted without sheath. In two patients with renal failure, the stenting was performed under echocardiography-guidance without contrast agent. The follow up showed a preserved function of the left ventricle in all patients. No relevant gradient was reported and no stent re-intervention was required. Sonographic follow up showed a patent femoral artery in all patients. Two patients were operated 73 and 110 days after stenting, and the stents were successfully removed. In the third patient the intervention was performed 130 days ago and he is waiting for the operation.

**Conclusion:**

CoA-stenting in VLWB and ELWB is feasible and can bridge them to the next surgery without complications. Echocardiography-guided CoA-stenting in VLWB is a considerate option especially in patients with renal failure. Accessing the femoral artery by experienced doctors, using local anesthesia before the puncture and before removing the sheath might help to protect the vessel from stenosis or occlusion.

## Introduction

Although the early repair of aortic coarctation in newborns is feasible with good outcome (1–3), some patients with CoA remain unamenable to be corrected in the neonatal period due to various reasons like impaired left ventricular function, very low birth weight, prematurity and interventricular hemorrhage. Treatment with prostaglandin E1 remains the first choice to keep the duct open for bridging the patients to the surgical repair. Transcatheter Balloon angioplasty of critical aortic coarctation in very low weight babies using either the umbilical artery or a surgical vessel access was reported (4–6). However, the high rate of recoarctation after angioplasty and need for re-intervention was observed (7). Stenting of CoA in newborns using pre-mounted coronary stents became a safe alternative to treat the acute symptoms and to bridge the patients to the surgical repair (8–10). However, stenting of CoA in very low weight babies (VLWB) < 1,500 g and extremely low weight babies (ELWB) < 1,000 g remains challenging due to the very small size of femoral artery, which makes accessing the femoral artery difficult and increases the risk of arterial spasm and arterial occlusion. The use of contrast agent in such patients can lead to a renal failure.

In this report, we aim to review the results of our center in CoA-Stenting in newborns with low and extremely low weight and to highlight on the role of echocardiography-guided stenting in such patients to avoid contrast agent especially in patients with renal insufficiency.

## Patients and methods

Three patients with low birth weight and isolated critical CoA were recruited between January 2020 and 2022 (Table 1). The emergency indication for intervention was first, to relieve the highly impaired LV function in all patients, second to improve the renal perfusion by renal failure in two patients and third to reduce the side effects of prostaglandin E1 in one patient.

**Table 1:** Summary table presenting patient characteristics, size of sheath and the follow up.

	**Patient 1**	**Patient 2**	**Patient 3**
Weight (g)	1,350	1,150	600
Length (cm)	40	45	33
Age (days)	30	12	15
GA^*^ (weeks)	32	28	28
IVH^**^ before the intervention	Yes	No	Yes
Coronary stent	Integrity	Integrity	Integrity
Size of stent (mm)	4/9	4/9	4/9
Vascular access	FA^***^	FA	FA
Sheath (French)	No sheath	4F	4F
FA stenosis/Occlusion	No	No	No
Pre-interventional RI^∧^	No	Yes	Yes
Angiography	Yes	No	No
Repair + remove of stent	Yes	Yes	Planned
Weight at operation (g)	2,600	3,800	
Age at operation (day)	73	110	>150^§^
Contrast agent	Yes	No	No

* GA, Gestational age;

** IVH, intracranial Hemorrhage;

*** FA, femoral artery;

∧ RI, renal insufficiency.

§ CoA stenting performed since 150 days and this patient waits for the operation.

Echocardiography, sonography of both femoral arteries and head ultrasound were recorded before and after the intervention and during follow up.

Echocardiography-guided intervention was done in two patients without using contrast agent.

The femoral artery was used as a vascular access for stenting. Local anesthesia with Mecain^®^ (10 mg/ml) 1 ml/Kg was performed before and after the intervention. Puncture was done under ultrasound-guidance in all patients. A 24 Gauge needle for accessing the artery and 0.14 wire for introducing the short sheath in the femoral artery and the coronary stent (steerable guidewire Wizdom™) were used. Stenting of the aortic coarctation was performed using of pre-mounted coronary stents (Medtronic Integrity coronary stents system) in all three patients.

## Description of patients

### Patient one

Premature with a gestational age (GA) of 32 weeks. Birth weight was 1,350 g. He had an isolated critical CoA with opened ductus under Prostaglandin E1. The indication of CoA-Stenting was a severe impaired LV function and repeated occurrence of imminent Necrotizing Enterocolitis (NEC).

### Patient two

Birth weight was 1,150 g [GA 32 weeks]. The indication of CoA-Stenting was severe impaired LV function. The duct was restricted, and the patient had associated renal insufficiency.

### Patient three

Birth weight 600 g, GA 28 weeks. The baby was intubated. The duct was restricted despite the Prostaglandin which was substituted with high doses (50 ng/Kg/d). The baby suffered from Prostaglandin related side effects. LV function was impaired.

## Results

Median weight at the intervention was 1,200 g (600–1,350), median length 40 cm (33–45 cm) and median base surface area. Median Age at intervention was: 15 day (12–30 days), median gestational age (GA) was 28 weeks (28–32). All interventions were successful without complications. One patient (patient three) was intubated before the intervention due to prostaglandin related apnoea and he was able to be extubated after stenting.

The stent was inserted without sheath in patient one. 4 F sheaths were used to insert the stents in the rest of patients.

Due to associated renal insufficient in two patients, the intervention was carried out successfully under transthoracic echocardiography without the use of angiography.

The left ventricular function (LV function) was relieved in all patients 24 h and was normalized 1 week after the intervention. Significant reduction of the gradient in region of isthmus after stenting was documented (residual gradient 12, 10, and 8 mmHg, respectively).

Renal parameter normalized in patient two and three and the renal failure was completely receded.

Follow up 1, 2, and 4 weeks after intervention showed a patent femoral artery in all three patients. Two patients were operated (33 and 65 days after the intervention) and the stent was successfully removed without problems. In the third patient (Patient three) the operation is planned at weight > 2.5 Kg.

## Discussion

Only a few studies about CoA-stenting in VLWB < 1,500 g (11) and in ELWB < 1,000 g (9, 12) were published in the last 10 years due to a very small number of patients underwent stenting of aortic coarctation in this very special population. The pre-mounted coronary stent was used for CoA-stenting in all studies included patients weighting under 1,500 g. One case report (13) about use and limitation Magmaris? bioresorbable coronary stent for CoA stenting in patient weighting 1,980 g showed a significant early stent failure due to a loss of radial force. The systemic sirolimus level detected 48 h after the intervention was high (5 ng/ml) and such level can increase the risk of occurrence of sirolimus-induced immunosuppression in very low weight babies.

**Figure 1:**
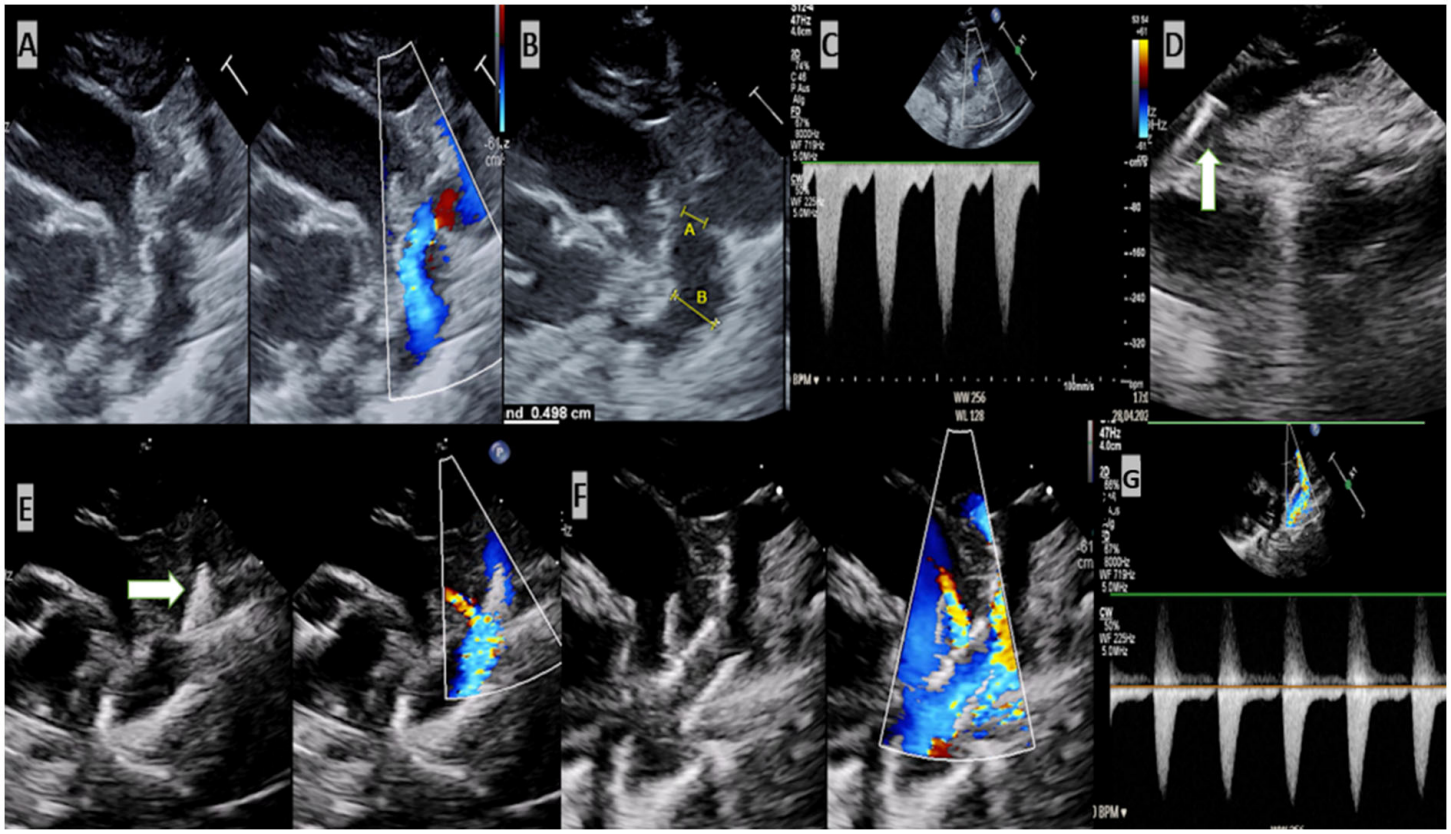
Echocardiogram-guided stenting of critical aortic coarctation. **(A)** Critical coarctation of aorta. **(B)** Measurement required for intervention [**(A)** the diameter of the stenosis and **(B)** is the diameter of the descending aorta]. **(C)** Increased velocity of blood in the region of stenosis. Insertion of the wire in the descending aorta. **(D)** Stent was positioned in the isthmus. **(E)** Stent was implanted. **(F,G)** Normal velocity of blood after implantation of stent.

In a previous study included five patients and published by Stegeman et al. (9) the stenting was performed successfully and without complications. This result is in agreement with ours, which showed a rapid recovery of the impaired organs after the intervention. The LV function was preserved, and the renal failure receded. However, our study is the first to report about a successful echocardiographic-guidance stenting of aortic coarctation without using contrast agent or angiography in severely ill VLWB and ELWB, suffering from renal insufficient. In two of our patients, the echocardiography provided excellent informations about the aortic arch, stenosis, PDA and the distance between left subclavian artery and the stenosis. Performing the necessary measurements to choose the suitable stent and introducing of the wire and stent as well as the implantation of the stent under guidance of echocardiography was feasible, practical and uncomplicated (Figure 1, supplementary material). However, we should not forget that echocardiography can miss a small intervention related dissection or a small aneurysm, due to its reduced resolution.

The use of the subclavian artery as a vascular access for CoA-stenting in a patient weighting 1,200 g was reported (14). Another study about surgical cut-down of the carotid artery in 42 patients weighting between 1.1 and 12.2 kg was published in 2016 (15) (two patients weighting under 1,500 g). The follow up showed a patent carotid artery in 38 patients underwent interventions in the aortic valve, PDA, aortic coarctation and Blalock Taussig Shunt. However, evidence of stenosis and thrombosis of the carotid artery was documented in three patients weighting between 1.7 and 2.1 Kg. Hematoma was documented in two patients and one patient had to be operated due to a pseudoaneurysm. In our cohort, we have chosen the femoral approach as vascular access to avoid any possible injury in the head and arm arteries in very low and extremely low weight babies.

In contrast to the previous study in which the accessed femoral arteries were occluded in all patients after the intervention, the follow up in our study showed that the femoral arteries in our cohort were patent without sonographic evidence of stenosis or another injury. This result might be explained with three points. Firstly, all femoral arteries were accessed by highly experienced personnel. Secondly, local anesthesia (1 ml/Kg) was used in all patients before the punctures and before removing the sheath to avoid any possible arterial spasm that can be caused by a very small pain-threshold. The third point is the administration of an adequate dose of heparin during and after the procedure which can protect the accessed vessel from thrombosis.

According to a study published in 2018 (16), the results showed that the incidence of intraventricular hemorrhage increased in VLWB with native aortic coarctation. This is in agreement with our observation which showed that 2 out 3 three patients in our population had IVH before the intervention. In sonographic follow up the IVH was reabsorbed in patient one but needed ventriculo-peritoneal shunt in patient two (4 weeks after CoA-stenting).

## Conclusion

Stenting of aortic coarctation in very low and extremely low weight babies is feasible and can bridge them to the surgical repair without significant gradient. Performing the intervention under echocardiography-guidance is uncomplicated and helps to avoid using the contrast agent in premature babies who suffer from renal failure. The femoral arteries might be preserved by accessing the arteries by experienced persons and by using local anesthesia to help avoiding spasm caused by pain.

## Data availability statement

The original contributions presented in the study are included in the article/Supplementary materials, further inquiries can be directed to the corresponding author/s.

## Ethics statement

Written informed consent was obtained from the individual(s) for the publication of any potentially identifiable images or data included in this article.

## Author contributions

Data collection, data analysis, and drafting the article: NM. Critical revision of the article: MS and PZ. Final approval of the version to be published: all authors.

## Conflict of interest

The authors declare that the research was conducted in the absence of any commercial or financial relationships that could be construed as a potential conflict of interest.

## Publisher's note

All claims expressed in this article are solely those of the authors and do not necessarily represent those of their affiliated organizations, or those of the publisher, the editors and the reviewers. Any product that may be evaluated in this article, or claim that may be made by its manufacturer, is not guaranteed or endorsed by the publisher.

## Supplementary material

Supplementary Video 1 Demonstration of the critical aortic coarctation.

Supplementary Video 2 Insertion of the wire in the descending aorta.

Supplementary Video 3 The coronary stent was introduced and positioned in the isthmus.

Supplementary Video 4 Inflation of the balloon of the pre-mounded coronary stent under echocardiography guidance.

Supplementary Video 5 Stent after implantation with good result.

Supplementary Video 6 Demonstration of the aortic arch, left subclavian artery, and implanted stent after stent implantation.
